# Carvedilol Attenuates the Progression of Hepatic Fibrosis Induced by Bile Duct Ligation

**DOI:** 10.1155/2017/4612769

**Published:** 2017-03-15

**Authors:** Xiaopeng Tian, Chunhong Zhao, Jinbo Guo, Shurui Xie, Fengrong Yin, Xiaoxia Huo, Xiaolan Zhang

**Affiliations:** ^1^Department of Gastroenterology, The Second Hospital of Hebei Medical University, Hebei Key Laboratory of Gastroenterology, Hebei Institute of Gastroenterology, Shijiazhuang, China; ^2^Department of Gastroenterology, Xingtai People's Hospital, Xingtai, Hebei 054000, China; ^3^Department of Fever Related Disease, The PLA General Hospital, Beijing 100853, China

## Abstract

*Background.* The sympathetic nervous system (SNS) is responsible for hepatic stellate cells (HSCs) activation and the accumulation of collagen that occurs in hepatic fibrogenesis. Carvedilol has been widely used for the complication of hepatic cirrhosis in the clinic. Furthermore, it has powerful antioxidant properties. We assessed the potential antifibrotic effects of carvedilol and the underlying mechanisms that may further enhance its clinical benefits.* Methods.* Using a bile duct ligation rat model of hepatic fibrosis, we studied the effects of carvedilol on the fibrosis, collagen deposition, and oxidative stress based on histology, immunohistochemistry, western blot, and RT-PCR analyses.* Results.* Carvedilol attenuated liver fibrosis, as evidenced by reduced hydroxyproline content and the accumulation of collagen, downregulated TIMP-1 and TIMP-2, and upregulated MMP-13. MMP-2 was an exception, which was decreased after carvedilol treatment for 2 weeks and upregulated after carvedilol treatment for 4 weeks. Carvedilol reduced the activation of HSCs, decreased the induction of collagen, transforming growth factor-*β*1, and MDA content, and strengthened the SOD activity. The antifibrotic effects were augmented as dosages increased.* Conclusions.* The study indicates that carvedilol attenuated hepatic fibrosis in a dose-dependent manner. It can decrease collagen accumulation and HSCs activation by the amelioration of oxidative stress.

## 1. Introduction

Hepatic fibrosis, a reversible wound-healing response to a variety of stimuli, is characterized by the accumulation of extracellular matrix (ECM) mainly secreted by activated hepatic stellate cells (HSCs) [1]. In normal liver, ECM is a highly dynamic substratum with a precisely regulated balance between synthesis and degradation. When ECM production exceeds degradation, hepatic fibrosis develops as a result of the progressive thickening of fibrotic septae and chemical cross-linking of collagen [2]. Transforming growth factor-*β*_1_ (TGF-*β*_1_) is the most potent fibrogenic cytokine in the liver, which is stored as an inactivated protein bound to a latency-associated protein. Once activated, TGF-*β*_1_ binds to Smad proteins, which enhances the transcription of target genes, including procollagen I and procollagen III, and then leads to fibrosis development [3].

Oxidative stress also plays a major role in hepatic fibrogenesis, which is an important stimulus to HSCs activation [4]. Reactive oxygen species (ROS) generated by oxidative stress can induce hepatocyte necrosis/apoptosis and HSCs activation [5]. In addition, ROS leads to a decrease in antioxidant defense such as superoxide dismutase (SOD). Besides being used as a nonselective *β*-adrenergic blocker, carvedilol has been studied as an antioxidant [6, 7]. It has been demonstrated that inhibition of ROS-induced Smad3 activation by carvedilol could improve myocardial fibrosis [8]. Moreover, carvedilol has been studied in the field of hepatic fibrosis. For cirrhotic rats, one-week carvedilol administration could decrease their portal pressure and endothelial-related vasodilatory activity [9]. Recently, a study by Hamdy and El-Demerdash has proved that carvedilol has potential antifibrotic effects on carbon tetrachloride (CCl_4_) induced liver fibrosis, which can be explained by amelioration of oxidative stress [10]. In conclusion, carvedilol as a nonselective *β*-adrenoreceptor antagonist and antioxidant might be beneficial for hepatic fibrosis treatment.

In the present study, hepatic fibrosis model was established by bile duct ligation (BDL) in Sprague-Dawley (SD) rats. Carvedilol was administered in three dosages 48 hours after BDL for 2 weeks and 4 weeks, respectively. In addition to assessing the effects of carvedilol on liver fibrosis, we also investigated its potential mechanisms.

## 2. Materials and Methods

### 2.1. Animal Experiments and Drug Treatment

Fifty adult male Wistar rats weighing 250–300 g were obtained from the Experimental Animal Center of Hebei Medical University (permission number 705188). The research was conducted in accordance with the internationally accepted principles for laboratory animal use and care as found in the US guidelines (NIH publication #85-23, revised in 1985). The experiment was performed in compliance with the national ethical guidelines for the care and use of laboratory animals (certificate number 911102).

The rats were randomly divided into five groups (10 rats per group): sham surgery control group, bile duct ligation (BDL) model group, low-dose carvedilol treatment group (0.1 mg·kg^−1^·d^−1^, CAR-L), medium-dose carvedilol treatment group (1 mg·kg^−1^·d^−1^, CAR-M), and high-dose carvedilol treatment group (10 mg·kg^−1^·d^−1^, CAR-H). Rat hepatic fibrosis model was established by applying BDL. Forty-eight hours after the operation, carvedilol was administered twice a day. Each group was also randomly divided into two groups, and their liver tissue specimens were separately taken 2 weeks and 4 weeks after BDL.

### 2.2. Liver Function Detection and Histological Examination

Blood was obtained from the left ventricular apex of rats and centrifuged at 3000*g* at 4°C for 10 min to collect the serum. The levels of alanine aminotransferase (ALT), aspartate aminotransferase (AST), total bilirubin (TBil), and albumin (Alb) were detected using a fully automatic biochemical analyzer. Liver specimens were fixed for 12–24 h in 4% phosphate-buffered paraformaldehyde (Huarui Scientific & Technological Co.) and then embedded in paraffin for light microscopy examination. Tissue sections (4 *μ*m thick) were stained with hematoxylin and eosin (H&E) for morphological evaluation and Masson trichrome (MT) for assessing the degree of fibrosis.

### 2.3. Immunohistochemical Staining

The 3 *μ*m thick liver tissues embedded in paraffin were deparaffinized and serially dehydrated in ethanol. Then, they were treated with 5% hydrogen peroxide for 10 min to inactivate the endogenous peroxidase. After 20 min of blocking with PBS containing 10% normal goat serum, the sections were incubated with primary antibodies. The primary antibodies included type I, III, and IV collagen (rabbit polyclonal antibody; Beijing Biosynthesis Biotechnology) used as a marker for collagen expression; smooth muscle actin polyclonal antibody (rabbit polyclonal antibody; Santa Cruz Biotechnology) used as a marker of activated HSC; TGF-*β*_1_ (rabbit polyclonal antibody; Beijing Biosynthesis Biotechnology) used as marker of cytokine. After treatment with HRP-conjugated goat anti-mouse IgG and 50 *μ*L of streptavidin-peroxidase solutions for 30 min at RT, the sections were stained with DAB and counterstained with hematoxylin. The positive areas showed the color of brown yellow. Immunohistochemical analysis was performed with Image-Pro Plus.

### 2.4. Hydroxyproline Determination

Collagen was detected by estimating the hydroxyproline content, an amino acid characteristic of collagen. Hepatic hydroxyproline was measured using a hydroxyproline detection kit (Jiancheng Institute of Biotechnology, Nanjing, China) according to the manufacturer's instructions.

### 2.5. Western Blot Analysis

Liver samples were homogenized with lysis buffer (Cell Signal Technology Inc., Danvers, MA) and centrifuged at 20,000 ×g for 60 minutes at 4°C. The resultant supernatants were used as the total liver protein and subjected to western blotting. The protein concentrations were determined by Bradford's method. Proteins were resolved by sodium dodecyl sulphate polyacrylamide gel electrophoresis and transferred to a polyvinyl difluoride membrane. Each blot was treated with the anti-b-actin antibody (*β*-actin 1 : 5,000; mouse monoclonal antibody; Sigma-Aldrich, St. Louis, MO), anti-matrix metalloproteinase-13 (MMP-13, 1 : 400), antitissue inhibitors of metalloproteinase-1 (TIMP-1, 1 : 400), anti-MMP-2 (1 : 300), and anti-TIMP-2 (1 : 400). All the above were from Santa Cruz Biotechnology. Then, secondary antibody (Santa Cruz Biotechnology) was added, and the reaction bands of western blot were quantified by Quantity One software (Bio-Rad Laboratories, Inc., Berkeley, CA) and modified by the *β*-actin. The values (% of control) are given as means ± SD of 5 animals.

### 2.6. Determination of Oxidative Damage and Antioxidant Enzyme Activity

Frozen liver tissues from each rat were defrosted and poached in ice-cold phosphate buffer. 100 mg liver tissue was weighed for homogenizing and centrifuged at 3,000 rpm for 10 min and the supernatant was imbibed for determination. Oxidative stress was determined by detecting the concentration of malonyldialdehyde (MDA) by thiobarbituric acid reactive substances. The experiment was conducted according to the manufacturer's instructions (Jiancheng Institute of Biotechnology, Nanjing, China). The conjugation was accompanied by an increase in absorbance at 532 nm. The cytosolic SOD activity was assayed using the SOD detection kit (Jiancheng Institute of Biotechnology, Nanjing, China). The autooxidation rate of epinephrine, which was progressively inhibited by the increasing amounts of SOD in the homogenate, was monitored spectrophotometrically at 550 nm. The amount of enzyme that inhibited 50% of epinephrine autooxidation was defined as 1 U of SOD activity.

### 2.7. Statistical Analysis

Data were analyzed statistically by one-factor analysis of variance (ANOVA). Differences between experimental groups were analyzed using Student-Newman-Keuls (SNK) multiple-range test. The significant level was set at *P* < 0.05. Results are expressed as means ± SD.

## 3. Results

### 3.1. Carvedilol Attenuated Liver Injury and Fibrosis

**Figure 1:**
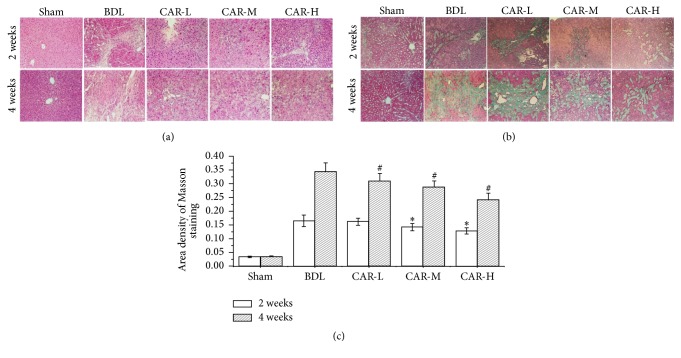
Representative photomicrographs of liver sections stained with H&E and MT, respectively. (a) H&E staining showed the pathologic changes in sham, BDL, CAR-L, CAR-M, and CAR-H groups two weeks and four weeks after bile duct ligation, respectively (magnification ×200). (b) MT staining showed the scar formation in the above five groups, respectively (magnification ×200). (c) The area density of Masson staining was significantly lower after carvedilol treatment. ^*∗*^*P* < 0.05* versus* BDL group for 2 weeks; ^#^*P* < 0.01* versus* BDL group for 4 weeks. CAR-H* versus* CAR-L, *P* < 0.05.

We found that the liver functions in hepatic fibrosis model groups were obviously damaged according to the expression of ALT, AST, TBil, and Alb. And the levels of ALT and AST in CAR-H groups were significantly lower than in model groups (Tables 1 and 2). H&E and MT staining were used to evaluate the therapeutic effects of different dosages of carvedilol on hepatic fibrosis. Two weeks after BDL, the liver tissues in BDL group exhibited distortion architecture, showing hepatocellular degeneration with formation of fibrous tissue infiltrated with inflammatory cells (Figure 1(a)). Four weeks after BDL, the increased hyperplasia of fibrous tissue led to fibrous septa formation (Figure 1(a)). Liver specimens obtained from carvedilol-treated group displayed a significant decrease in fibrous tissue and the hepatocytes largely retained their normal appearance, especially in the CAR-H group (Figures 1(a) and 1(b)). As determined by Masson's trichrome staining, BDL markedly induced liver fibrosis. These alterations were significantly attenuated by carvedilol administration, and the efficacy was more obvious as the dosages increased (Figure 1(c)).

### 3.2. Carvedilol Prevented the Abnormal Collagen Deposition

**Figure 2:**
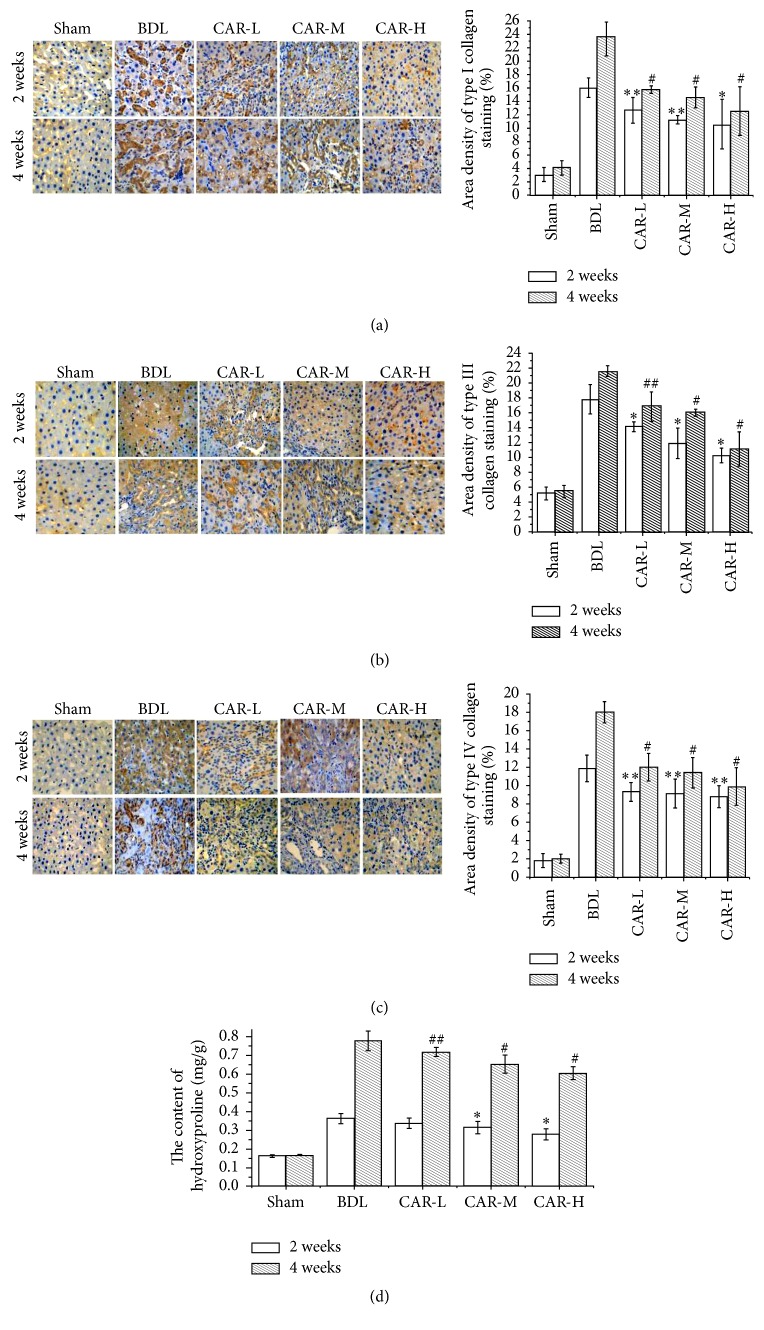
ECM-related collagen I, collagen III, and collagen IV expression and hydroxyproline content in the five groups. (a) Expression of collagen I by immunohistochemical staining (magnification ×200). (b) Expression of collagen III by immunohistochemical staining (magnification ×200). (b) Expression of collagen IV by immunohistochemical staining (magnification ×200). (a–c) Expressions of collagen I, collagen III, and collagen IV were the highest in the BDL group, which were decreased in association with the carvedilol dosage. ^*∗∗*^*P* < 0.05, ^*∗*^*P* < 0.01* versus* BDL group for 2 weeks; ^##^*P* < 0.05, ^#^*P* < 0.01* versus* BDL group for 4 weeks. (d) Liver hydroxyproline content expressed as *μ*g/mg of wet tissue. The content of hydroxyproline was markedly decreased after carvedilol treatment, especially in the CAR-M and CAR-H groups. ^*∗*^*P* < 0.01* versus* BDL group for 2 weeks; ^##^*P* < 0.05, ^#^*P* < 0.01* versus* BDL group for 4 weeks. CAR-H* versus* CAR-L, *P* < 0.05.

Type I, III, and IV collagen expressions were detected by immunohistochemistry staining. It was demonstrated that the expressions of type I, III, and IV collagens in carvedilol-treated groups were markedly reduced compared to BDL group, while no marked differences were found among the three carvedilol-treated groups (Figures 2(a), 2(b), and 2(c)).

Hydroxyproline is unique to collagen and thus serves as a specific biochemical marker of collagen production. Two weeks after BDL, the hydroxyproline content in BDL group was markedly higher than that in the sham group. However, it was significantly decreased in CAR-M and CAR-H groups (0.315 ± 0.034 *μ*g·mg^−1^, 0.279 ± 0.031 *μ*g·mg^−1^* versus *0.363 ± 0.027 *μ*g·mg^−1^; *P* < 0.01, *P* < 0.01, resp.) (Figure 2(d)). Four weeks after BDL, the hydroxyproline content in the CAR-L, CAR-M, and CAR-H groups was significantly lower than that in BDL group (0.719 ± 0.024 *μ*g·mg^−1^, 0.654 ± 0.048 *μ*g·mg^−1^, and 0.605 ± 0.034 *μ*g·mg^−1^* versus *0.778 ± 0.052 *μ*g·mg^−1^; *P* < 0.05, *P* < 0.01, and *P* < 0.01, resp.) (Figure 2(d)). And a significant difference was found between the CAR-H and CAR-L groups (*P* < 0.05).

### 3.3. Carvedilol Inhibited HSC Activation and Induced Activated HSC Apoptosis

**Figure 3:**
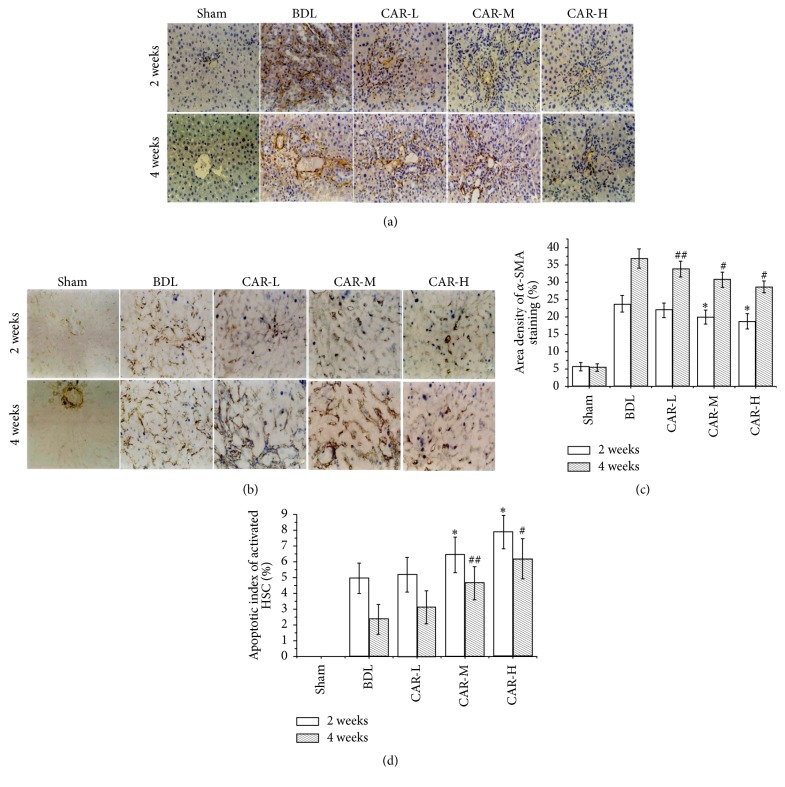
*α*-SMA expression and the apoptotic index of activated HSC. (a, c) Expression of *α*-SMA by immunohistochemical staining (magnification ×200). Treatment with carvedilol was effective in inhibiting HSC activation. Moreover, it was related to the doses of the drug. ^*∗*^*P* < 0.01* versus* BDL group for 2 weeks; ^##^*P* < 0.05, ^#^*P* < 0.01* versus* BDL group for 4 weeks. CAR-M* versus* CAR-L, *P* < 0.05; CAR-H* versus* CAR-M, *P* < 0.05. CAR-M* versus* CAR-L, *P* < 0.05; CAR-H* versus* CAR-M, *P* < 0.05. (b, d) Apoptosis of activated HSC by TUNEL and *α*-SMA immunohistochemical double staining. The apoptosis of activated HSC was markedly increased after carvedilol treatment, especially in the CAR-M and CAR-H groups. ^*∗*^*P* < 0.05, ^*∗*^*P* < 0.01* versus* BDL group for 2 weeks; ^##^*P* < 0.05, ^#^*P* < 0.01* versus* BDL group for 4 weeks. CAR-H* versus* CAR-M, *P* < 0.05.

We investigated the changes of HSCs in the present BDL rats to examine the effect of BDL-induced overactive sympathetic tone on the activation of HSCs, the key cell for the progressive accumulation of collagen in fibrosis disease. Immunohistochemistry staining for a-SMA, the marker of the activated HSCs, was mildly increased in the BDL group compared to that in sham group and was reduced by carvedilol treatment. After the 2-week carvedilol administration, the *α*-SMA expression in CAR-M and CAR-H groups was markedly reduced compared to that in BDL group (19.95  ±  2.02%, 18.77  ±  2.15%* versus* 23.77  ±  2.43%; *P* < 0.01, *P* < 0.01, resp.) (Figures 3(a) and 3(c)). After the 4-week carvedilol administration, the positive expression of *α*-SMA in CAR-L, CAR-M, and CAR-H groups was significantly decreased compared to that in BDL group (33.86 ± 2.23%, 30.77 ± 2.17%, and 28.72 ± 1.74%* versus *36.88% ± 2.83%; *P* < 0.05, *P* < 0.01, and *P* < 0.01, resp.) (Figures 3(a) and 3(c)). And a significant difference was found among the carvedilol-treated groups (CAR-M* versus* CAR-L, *P* < 0.05; CAR-H* versus* CAR-M, *P* < 0.05).

HSCs apoptosis was determined by TUNEL and *α*-SMA immunohistochemical double staining. Among the 2-week groups, the apoptosis index of HSC was markedly higher in the CAR-M and CAR-H groups than in BDL group (6.45 ± 1.12%, 7.89 ± 1.06%* versus *4.95 ± 0.95%; *P* < 0.05, *P* < 0.01, resp.) (Figures 3(b) and 3(d)). Similarly, in the 4-week groups, the apoptosis index was markedly higher in the CAR-M and CAR-H group than in BDL group (4.63  ±  1.06%, 6.17 ± 1.27%* versus* 2.35  ±  0.94; *P* < 0.05, *P* < 0.01, resp.) (Figures 3(b) and 3(d)). And a statistical difference was found between the CAR-H group and CAR-M groups (*P* < 0.05). The results suggested that carvedilol may inhibit the activation of HSCs and promote apoptosis via the antisympathetic predominance pathway.

### 3.4. The Effects of Carvedilol on MMPs and TIMPs

**Figure 4:**
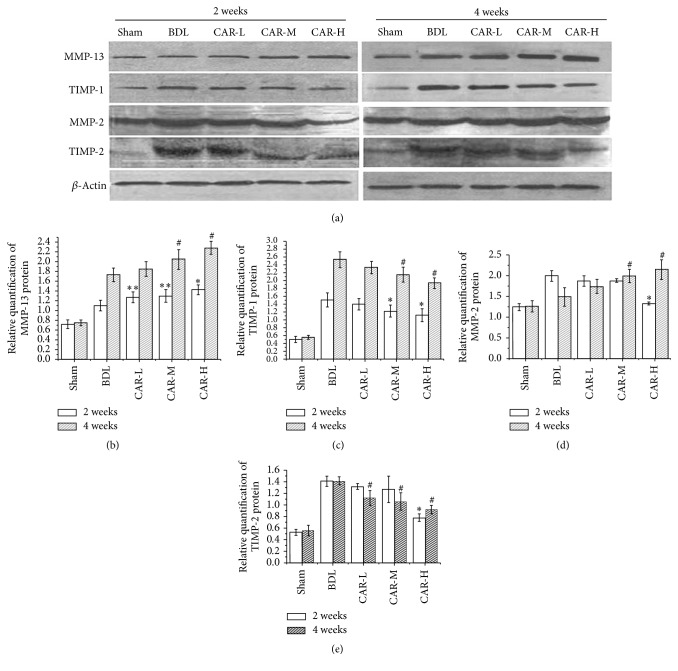
Expression of MMP-13, TIMP-1, MMP-2, and TIMP-2 by western blot analysis. (a) Band of each test index detected by western blot. (b) The expression of MMP-13 was markedly increased after carvedilol therapy. ^*∗∗*^*P* < 0.05, ^*∗*^*P* < 0.01* versus* BDL group for 2 weeks; ^#^*P* < 0.01* versus* BDL group for 4 weeks. (c) TIMP-1 expression was significantly decreased after carvedilol treatment, especially in the CAR-M and CAR-H groups. ^*∗*^*P* < 0.01* versus* BDL group for 2 weeks; ^#^*P* < 0.01* versus* BDL group for 4 weeks. (d) MMP-2 expression was significantly decreased after carvedilol treatment for 2 weeks in the CAR-H group, while no obvious changes were found among the BDL, CAR-L, and CAR-M groups. ^*∗*^*P* < 0.01* versus* BDL group. The expression of MMP-2 was increased after carvedilol treatment for 4 weeks, especially in the CAR-M and CAR-H groups. ^#^*P* < 0.01* versus* BDL group. (e) TIMP-2 expression was significantly decreased after carvedilol treatment for 2 weeks in the CAR-H group, while no obvious changes were found among the BDL, CAR-L, and CAR-M groups. ^*∗*^*P* < 0.01* versus* BDL group. The expression of TIMP-2 was also decreased after carvedilol treatment for 4 weeks. ^#^*P* < 0.01* versus* BDL group.

The degradation of the matrix components markers MMP-2, TIMP-2, MMP-13, and TIMP-1 was used for the same rats in order to evaluate the accumulation and the degradation of matrix components based on the western blot and real-time Q-PCR (Figure 4). Two weeks after BDL, western blot results showed that MMP-2 protein expression was markedly higher in BDL group than in sham group (1.99 ± 0.13* versus *1.24 ± 0.09; *P* < 0.01), but it was significantly reduced in CAR-H group (1.33 ± 0.04). After carvedilol treatment for 4 weeks, the MMP-2 protein expression in CAR-M and CAR-H group was significantly higher than that in BDL group (1.99 ± 0.16, 2.15 ± 0.24* versus *1.49 ± 0.22; *P* < 0.01, *P* < 0.01, resp.).

After carvedilol treatment for 2 weeks, the MMP-13 protein expression in CAR-L, CAR-M, and CAR-H groups was significantly higher than that in BDL group (1.27 ± 0.11, 1.30 ± 0.13, and 1.43 ± 0.1* versus *1.1 ± 0.11; *P* < 0.05, *P* < 0.05, and *P* < 0.01, resp.). Similar results were shown in 4-week carvedilol groups. And a significant difference was found between the CAR-L and CAR-H groups (*P* < 0.05). The MMP-2 and MMP-13 mRNA expressions showed the same tendency as the protein expression above (data was not shown).

TIMP-1 and TIMP-2, mainly produced by HSC, combine with active MMPs to inhibit their activity. Two weeks and 4 weeks after BDL, the protein expression of TIMP-1 and TIMP-2 in BDL group was increased compared with that in sham group. After BDL and carvedilol treatment for 2 weeks and 4 weeks, TIMP-1 protein expression in CAR-M and CAR-H groups was markedly reduced. In the 2-week groups, the protein expression of TIMP-2 in the CAR-H group was dramatically lower than that in the BDL group (0.78 ± 0.06* versus *1.41 ± 0.09; *P* < 0.01). In the 4-week groups, TIMP-2 in CAR-L, CAR-M, and CAR-H groups was significantly reduced compared with that in the BDL group (1.12 ± 0.13, 1.06 ± 0.15, and 0.92 ± 0.07* versus *1.42 ± 0.07; *P* < 0.01, *P* < 0.01, and *P* < 0.01, resp.) (Figures 4(a) and 4(e)). And there was a significant difference between the CAR-L and CAR-H groups (*P* < 0.01). The results determined by RT-PCR were the same as the results above (the data was not shown). All these results suggest that carvedilol may modify the shift in the balance of the accumulation and degradation of matrix components in the BDL rats.

### 3.5. Carvedilol Inhibited TGF-*β*_1_ Expression

**Figure 5:**
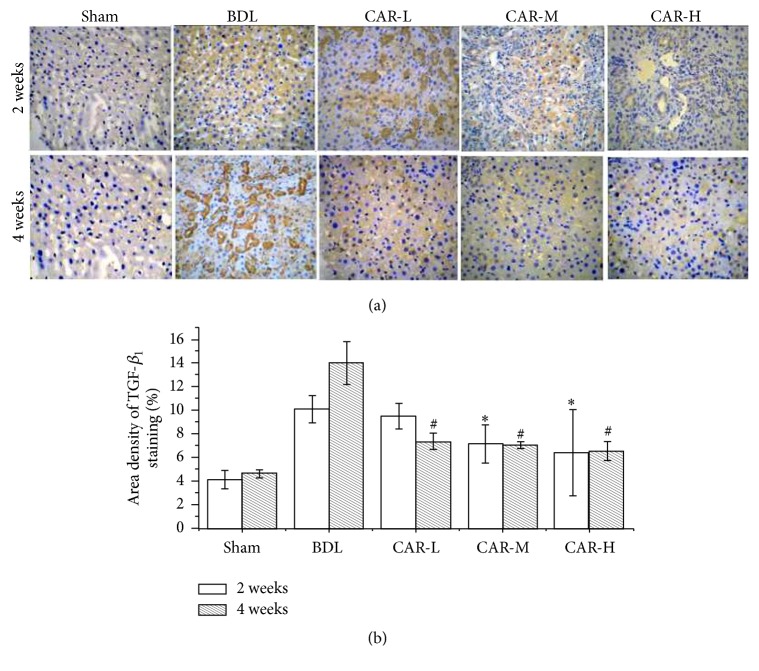
Expression of TGF-*β*_1_ by immunohistochemical staining (magnification ×200). The expression of TGF-*β*_1_ was significantly decreased after carvedilol therapy. ^*∗*^*P* < 0.01* versus* BDL group for 2 weeks; ^#^*P* < 0.01* versus* BDL group for 4 weeks.

We detected the expression of TGF-*β*_1_ by immunohistochemistry staining. In the 2-week groups, the expression of TGF-*β*_1_ in CAR-M and CAR-H groups was markedly reduced compared with that in the BDL group (7.14 ± 1.60%, 6.44 ± 3.64%* versus *10.10 ± 1.14%; *P* < 0.01, *P* < 0.01, resp.). But no statistical difference was found between the CAR-M and CAR-H groups. Four weeks after BDL, TGF-*β*_1_ expression in CAR-L, CAR-M, and CAR-H groups was significantly decreased compared with that in the BDL group (7.35 ± 0.68%, 7.04 ± 0.33%, and 6.53 ± 0.81%* versus *14.00 ± 1.81%; *P* < 0.01, *P* < 0.01, and *P* < 0.01, resp.) (Figures 5(a) and 5(b)).

### 3.6. Carvedilol Inhibited Lipid Peroxidation and Enhanced SOD Activities

**Figure 6:**
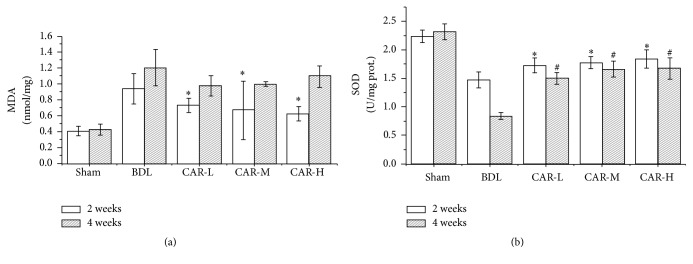
Effect of carvedilol on oxidative stress markers. (a) The MDA level was markedly decreased after carvedilol treatment for 2 weeks. ^*∗*^*P* < 0.01* versus* BDL group. However, no statistical differences were found compared with the BDL group after applying carvedilol in different doses for 4 weeks. (b) SOD activities were markedly higher after carvedilol treatment. ^*∗*^*P* < 0.01* versus* BDL group for 2 weeks; ^#^*P* < 0.01* versus* BDL group for 4 weeks.

As expected, two weeks after BDL, thiobarbituric acid method (TBA) showed that MDA contents in the CAR-L, CAR-M, and CAR-H groups were significantly lower than those in the BDL group (0.73 ± 0.09, 0.67 ± 0.62, and 0.63 ± 0.09* versus *0.94 ± 0.19; *P* < 0.01, *P* < 0.01, and *P* < 0.01, resp.), while no statistical difference was found among the carvedilol-treated groups. Four weeks after BDL, there was no significant difference between the carvedilol-treated groups and the BDL group (Figure 6(a)).

We also determined the SOD activities by xanthine oxidase method. Two weeks after BDL, it was shown that SOD activities in the CAR-L, CAR-M, and CAR-H groups were higher than those in the BDL group (1.73 ± 0.13, 1.78 ± 0.11, and 1.84 ± 0.16* versus *1.48 ± 0.14; *P* < 0.01, *P* < 0.01, and *P* < 0.01, resp.), while no marked differences were found among the three carvedilol-treated groups. Similarly, four weeks after BDL, SOD activities in the CAR-L, CAR-M, and CAR-H groups were higher than those in the BDL group (1.50 ± 0.10, 1.66 ± 0.14, and 1.67 ± 0.19* versus *0.84 ± 0.06; *P* < 0.01, *P* < 0.01, and *P* < 0.01, resp.) (Figure 6(b)).

## 4. Discussion

Carvedilol is a nonselective beta-blocker with potent antioxidant and free radical scavenging properties, which is used in the treatment of portal hypertension commonly associated with chronic liver diseases. Considering these powerful antioxidant properties of carvedilol, we predicted that carvedilol may exert an antifibrotic effect on chronic liver injury. This means carvedilol has additional clinical benefits. So, in the present study, we investigated the efficacy of carvedilol on BDL-induced hepatic fibrosis and the potential mechanisms involved. Three dosages of carvedilol treatment for 2 weeks and 4 weeks were used, which was beneficial for observing the curative effect on different stages of fibrosis.

In the study, H&E and Masson staining confirmed the established hepatic fibrosis model. The liver inflammatory and fibrous formations were markedly alleviated by carvedilol treatment. Hepatic fibrosis is characterized by abnormal accumulation of ECM, which is changed not only in quality but also in quantity. In normal liver, the low-density basement membrane-like matrix in the space of Disse is mainly composed of collagens IV and VI. However, the fibrillar collagens including collagens I and III and fibronectin are markedly increased in hepatic fibrosis [1]. This indicated that carvedilol diminished the expression of type I, III, and IV collagens in a dose-dependent manner. Determination of hydroxyproline content in liver tissues is regarded as a good method for quantifying fibrosis and evaluating the efficacy of new antifibrotic agents. In the study, the results implied that carvedilol administration significantly decreased the content of hydroxyproline. TGF-*β* is the mostly potent fibrogenic cytokine, which has three major isoforms (TGF-*β*_1_, TGF-*β*_2_, and TGF-*β*_3_). TGF-*β*_1_ is the principal isoform implicated in liver fibrosis. The activated TGF-*β*_1_ combines with Smad proteins via its cognate receptors, which enhances the transcription of target genes, including collagens I and III [11, 12]. The results confirmed that carvedilol administration strongly reduced TGF-*β*_1_ expression. Therefore, we inferred that the inhibition of TGF-*β*_1_ by carvedilol was one of the pathways for decreasing collagen expression.

HSCs are typically found in the space of Disse in a quiescent state [13]. HSCs may be activated to myofibroblasts expressing *α*-SMA by several stimuli such as cytokines and inflammatory mediators. Activated HSCs migrate to and proliferate in sites of liver injury, synthesize ECM components, and upregulate the expression levels of *α*-SMA and collagen matrices [14]. Hence, *α*-SMA is a marker of HSC activation and proliferation. The results of *α*-SMA expression showed that carvedilol administration inhibited HSC activation. Furthermore, *α*-SMA and TUNEL double staining demonstrated that carvedilol increased the apoptosis of activated HSC. Regulating the biologic behavior of HSC may be one of the reasons for carvedilol to prevent the progression of hepatic fibrosis.

Hepatic fibrosis occurs because of the synthesis and excessive deposition of ECM in the space of Disse along with insufficient ECM degradation. The equilibrium between MMPs and TIMPs mainly determines the homeostasis of ECM. MMPs are the main enzymes responsible for ECM degradation and TIMPs are their specific inhibitors [15]. In the early stage of hepatic fibrogenesis, MMP-2 would degrade the normal ECM around HSCs, which accelerates the HSCs activation [16]. In the late stage, MMPs including MMP-2 are beneficial to the treatment of hepatic fibrosis through degrading ECM. Activated HSCs upregulate the expressions of TIMP-1 and TIMP-2 [17]. TIMP-1 not only restrains the ability of MMPs, but also inhibits the apoptosis of activated HSCs [18]. In the study, carvedilol was shown to upregulate MMP-13 expression. Meanwhile, it downregulated the expressions of TIMP-1 and TIMP-2 in a dose-dependent manner. However, MMP-2 expression was different from MMP-13. After the two-week carvedilol treatment, MMP-2 expression was decreased compared with that in the BDL group, but it markedly increased after the 4-week carvedilol treatment. Hence, we speculated that carvedilol could prevent the activation of HSCs through restraining MMP-2 expression in the early stage of fibrogenesis. In the late stage, carvedilol upregulated the expressions of MMP-2 and MMP-13, which was beneficial for ECM degradation.

Bile duct ligation is an established method for investigating cholestasis and hepatic fibrosis, which leads to changes in the equilibrium between antioxidant and prooxidant activities [19]. The deposition of hydrophobic bile acids damages the mitochondrial electron transport chain and enhances the production of ROS. ROS plays an important role in amplifying the inflammatory response, stimulating the production of profibrogenic mediators, and initiating hepatic fibrogenesis. Therefore, inhibiting oxidative stress can hinder the progression of hepatic fibrosis. We investigated the influence of carvedilol on oxidative stress condition through determining MDA level and SOD activity. MDA is the main product of lipid peroxidation and its concentration is presented as the total level of lipid peroxidation products [20]. After carvedilol treatment for 2 weeks, the MDA level was markedly decreased compared with that in the BDL group, while no statistical difference was found among the three carvedilol-treated groups. Unexpectedly, after carvedilol treatment for 4 weeks, the MDA level was not decreased. This demonstrated that low dosage of carvedilol could decrease the level of MDA in the early stage of liver fibrosis. However, the antioxidant efficacy was decreased with the passage of time. As a free radical scavenging enzyme, SOD protects the biological systems from oxidative stress. The current study showed a distinct decrease of SOD activity in BDL-induced liver fibrosis. On the other hand, it was significantly increased after carvedilol treatment. Thus, we concluded that carvedilol reduced the oxidative stress through decreasing the MDA level and upregulating the SOD activity. The efficacy was more obvious in the early stage of fibrogenesis.

In summary, carvedilol plays a preventive role in a dose-dependent fashion in the progression of hepatic fibrosis induced by BDL, which is associated with decreased TGF-*β*_1_ expression, increased HSCs apoptosis, and increased ratio of MMPs/TIMPs, as well as its antioxidant property. Consequently, carvedilol treatment as early as possible in the maximum dosage that patients are able to bear may be beneficial for preventing hepatic fibrosis progression.

## Figures and Tables

**Table 1:** Levels of ALT, AST, Alb, and TBil in BDL model after carvedilol treatment for two weeks.

Group	ALT (U/L)	AST (U/L)	ALB (g/L)	TBil (*μ*mol/L)
Sham	45.8 ± 4.18	128.9 ± 15.12	36.1 ± 2.77	1.4 ± 0.37
BDL	92.6 ± 11.22^*∗*^	418.1 ± 40.71^*∗*^	27.5 ± 3.98^*∗*^	84.7 ± 11.39^*∗*^
CAR-L	90.6 ± 9.26^*∗*^	406.4 ± 34.53^*∗*^	27.3 ± 2.36^*∗*^	85.6 ± .9.21^*∗*^
CAR-M	86.5 ± 13.86^*∗*^	391.2 ± 45.51^*∗*^	28.1 ± 3.25^*∗*^	82.5 ± 12.24^*∗*^
CAR-H	81.7 ± 10.65^*∗*#^	370.3 ± 38.19^*∗*#^	28.4 ± 3.27^*∗*^	78.7 ± 13.12^*∗*^

BDL: bile duct ligation; sham: sham-operated; CAR: carvedilol; ALT: alanine transaminase; AST: aspartate transaminase; Alb: albumin; TBil: total bilirubin. ^*∗*^*P* < 0.05  *versus* sham; ^#^*P* < 0.05  *versus* BDL.

**Table 2:** Levels of ALT, AST, Alb, and TBil in BDL model after carvedilol treatment for four weeks.

Group	ALT (U/L)	AST (U/L)	ALB (g/L)	TBil (*μ*mol/L)
Sham	48.7 ± 6.24	136.9 ± 16.35	35.2 ± 3.12	1.39 ± 0.45
BDL	121.7 ± 15.33^*∗*^	603.9 ± 60.92^*∗*^	25.3 ± 5.06^*∗*^	141.2 ± 17.06^*∗*^
CAR-L	118.35 ± 14.93^*∗*^	586.32 ± 47.56^*∗*^	25.5 ± 4.12^*∗*^	135.6 ± 18.8^*∗*^
CAR-M	112.46 ± 16.89^*∗*^	552.1 ± 40.55^*∗*#^	26.09 ± 3.78^*∗*^	133.2 ± 16.32^*∗*^
CAR-H	105.2 ± 13.34^*∗*#^	519.6 ± 58.4^*∗*#^	27.1 ± 3.49^*∗*^	127.7 ± 13.06^*∗*^

BDL: bile duct ligation; sham: sham-operated; CAR: carvedilol; ALT: alanine transaminase; AST: aspartate transaminase; Alb: albumin; TBil: total bilirubin. ^*∗*^*P* < 0.05  *versus*  sham; ^#^*P* < 0.05  *versus*  BDL.
